# Is nursing shortage in Israel inevitable?

**DOI:** 10.1186/2045-4015-3-10

**Published:** 2014-03-25

**Authors:** Linda H Aiken, Matthew D McHugh

**Affiliations:** 1 418 Curie Blvd, Philadelphia, PA 19104-4217, USA

## Abstract

Israel has a low density of professional nurses. New evidence suggests less than optimal hospital work environments may undermine efficient and effective delivery of nursing care and contribute to job dissatisfaction and nurse turnover among nurses who are in short supply. Potential approaches to address these challenges are discussed.

## Introduction

Nursing shortages in developed countries have two related causes: 1) inadequate national/regional supply of nurses; and 2) in health care services, too few budgeted nurse positions and poor work environments that waste nursing resources. International evidence suggests that both of these causes of nursing shortages are remediable.

In this issue of the Israel Journal of Health Policy Research, DeKeyser-Ganz and Toren report on new research in Israel, a country with too few nurses, showing that hospitals have not done all they can to create optimal work environments to maximize the contributions of the nurses they have [1]. The convenience sample of hospitals they studied probably trended toward the better hospitals in Israel, and thus their findings that hospital work environments were not outstanding does not portend well for the rest of the hospitals in the country and the patients cared for in them. In addition to lackluster ratings on work environment features that are easy to improve and necessary for good patient outcomes such as nurse participation in hospital affairs, nurses gave their hospitals only average ratings on staffing adequacy — a measure that is highly correlated with actual patient to nurse staffing ratios and a variety of patient outcomes including preventable deaths [2]. The authors conclude that less than optimal hospital work environments are associated with less job satisfaction among nurses and greater tendency of nurses to consider leaving their positions thus potentially exacerbating a national nurse shortage.

### Nurse work environments are important for patients too

A substantial body of international research shows that inadequate nurse work environments are associated with poor patient outcomes. We surveyed large numbers of nurses and patients in 210 hospitals in 8 countries in Europe and 430 hospitals in the USA asking whether they would recommend their hospitals. Nurses based their assessments largely on the quality of the nurse work environment and staffing adequacy. Nurses and patients were in remarkably close agreement on which hospitals were good places to work and to get care. Indeed, we found that patient to nurse staffing ratios and nurses’ assessments of the quality of nurse work environment were significant predictors of patients’ global assessments of hospitals including patients’ overall hospital ratings and whether they would recommend the hospital to a family member or friend who needed hospital care [3]. Thus, work environment features that result in job dissatisfaction, burnout, and intent to leave among nurses are also associated with lower patient satisfaction with their care.

It is very common for required nursing care to be left undone because of lack of time, and missed care occurs significantly more often in hospitals with poor or mixed work environments [4]. The types of care most likely to be left undone are related to interpersonal communications between nurses and patients and their families including the provision of comfort measures, talking with patients, education of patients and families about their conditions and how to best manage them, and discharge planning—types of nursing care that convey nurses’ personal regard for patients. Attention by nurses to patient teaching and discharge planning are associated with fewer hospital readmissions as well, readmissions being a prime target for cost-savings [5].

In addition to patient satisfaction, inadequate nurse work environments in a variety of countries—Canada, China, Japan, New Zealand, South Korea, Thailand, and USA--have been found to be significantly associated with a range of less favorable quality of care outcomes including greater risk of mortality after surgery [2,6,7].

One component of a good hospital nurse work environment, as discussed by DeKeyser-Ganz and Toren, is staffing adequacy. Achieving adequate and safe patient to nurse ratios in hospitals is challenging when the national supply of nurses is low, as in Israel, and with constraints on health care expenditures which is the case in all countries. However, the other 4 domains measured by the Practice Environment Scale of the Nursing Work Index—nurse participation in hospital affairs, administrative support for nursing, collaborative relations between doctors and nurses, and a priority on quality improvement—reflect managerial commitments to a culture in which nurses can be efficient, effective, and successful. Indeed, evidence suggests that investments in improving nurse staffing have their greatest positive effects on improving patient safety and quality outcomes in hospitals with good work environments, and that investments in improved staffing in hospitals with poor environments is not a high value investment [2]. Changing culture is hard work but it is not expensive in monetary terms. Thus, improving nurse work environments is a relatively low-cost lever with substantial promise of improving nurse retention and patient outcomes. From a comparative effectiveness perspective, it makes good clinical and business sense to improve nurse work environments.

### Improving hospital work environments

In a rare longitudinal panel study of hospitals using the same measure of the nurse work environment used in Israel, researchers found substantially greater improvements in nurse satisfaction and reductions in burnout and intent to leave among nurses in hospitals that had implemented measureable changes in work environments between 1999 and 2006 than in hospitals where work environments had not improved [8]. Additional longitudinal research is needed to confirm a causal linkage between improvements in hospital work environments and improved nurse and patient outcomes to give greater confidence to hospital administrators that investments in improved work environments are likely to provide value to the institution.

Research on hospitals that have achieved Magnet designation by the American Nurses Credentialing Center (ANCC), a voluntary program open to hospitals in all countries with the aim of creating excellent work environments, also suggests that hospital work environments can be improved with resulting positive workforce and patient outcomes. Magnet hospitals have better nurse work environments and better nurse and patient outcomes, including lower mortality than matched hospitals [9]. The ANCC Magnet program has developed a blue print to guide hospitals in improving their work environments, and there is evidence that all kinds of hospitals, large and small, can be successful in improving their work environments [10]. In the USA, close to 10% of hospitals have achieved Magnet status, which has been accepted as a marker of a high performing institution by the business-led organization Leap Frog. While most Magnet hospitals are currently located in the USA, where the accreditation program began, an increasing number of countries can point to Magnet hospitals including regional neighbors Lebanon and Saudi Arabia. Whether or not hospitals elect to pursue Magnet status, the ANCC blueprint for Magnet and the substantial literature developed by Magnet hospitals are helpful guides for initiating work environment improvement.

### Is Israel’s nursing shortage solvable?

The paper by DeKeyser-Ganz and Toren points to the need to improve work environments in Israeli hospitals in order to retain in clinical care roles the nurses the nation has. Related research in other countries shows improvements in nurse work environments hold promise for utilizing nurses more efficiently and effectively to achieve better outcomes for patients. Hospital nurses are overworked but underemployed in the sense that they spend much of their time working around operational failures that have persisted in hospitals for decades and require nurses to undertake tasks that do not require their unique expertise. Operations engineers estimate that nurses are interrupted in mid-task on average once an hour to solve problems of missing or broken equipment, missing drugs and supplies, wrong meals delivered, missing lab tests, and obtaining supplies like blood from departments that do not operate on a 24-hour basis [11]. In no other industry where the stakes are so high would such systems failures be acceptable and common. Results like this underscore the need for nurses to be involved in hospital decision-making to get these problems solved. A country like Israel with too few nurses cannot afford to waste the time of those it does have.

Israel has 4.97 nurses per 1000 population while the average for the 40 Organisation for Economic Co-operation and Development (OECD) countries including Israel is 8.4 nurses per 1000. Israel was one of only two OECD countries to experience an average annual negative growth rate in nurses between 2000 and 2009. Also noteworthy, but not often mentioned in the context of the nursing shortage, Israel has a ratio of only 1.3 nurses to each doctor in comparison to the OECD average of 2.8 nurses per doctor, and the World Health Organization’s recommended ratio of 3-4 nurses per doctor in developed countries [12]. Israel is overproducing doctors relative to its supply of nurses, even though the physician to population ratio has been declining in Israel both in absolute terms and relative to the OECD average. While the roles of doctors and nurses overlap, and nurses have safely assumed some roles provided previously by doctors, there are few examples of qualified doctors providing needed nursing care; nor is this desirable since doctors and nurses have different education, knowledge, and expertise. Thus having an imbalance in the ratio of nurses to doctors is not useful in addressing nursing shortages, and may exacerbate the nurse shortage by diminishing the potential applicant pool for nursing. If there is a perceived shortage of primary care physicians, using the opportunity to expand opportunities for nurses might stimulate more interest in nursing as a career choice.

Prerequisites for having enough nurses include the availability of good jobs and professionally rewarding working conditions and career opportunities. DeKeyser-Ganz and Toren’s research suggest that there are neither enough jobs for nurses in hospitals since workloads are high, nor professionally rewarding enough working conditions. Both of these limitations—not enough jobs and not good enough professional working conditions—need to be solved before enough Israelis will be interested in becoming nurses.

Other changes, more cultural in nature, are also needed as reflected in national interest in medicine as a career in Israel but not so much in nursing. The USA is a good example of how nursing can be transformed into a more attractive career choice with startling results. Nursing in the USA is now among the most popular career choices with over 30,000 qualified nurse applicants turned away from admission to nursing schools each year. Graduations from nursing schools have doubled over 20 years, and most nurses pay for their own education. Many nursing school applicants have university degrees in other fields, and are beginning new careers in nursing. The Gallup public opinion poll consistently shows that nurses rank highest in the public’s trust among all occupations including doctors. The Institute of Medicine of the National Academy of Sciences recently called for nurses to have expanded roles in clinical care and leadership roles in redesigning and managing health care services. The shift in nurses’ prospects in the USA came about as a result of national efforts to more accurately portray to the public through mass media the importance of nurses. Informing the public about nurses’ contributions to their health and safety was helped by the establishment of the National Institute of Nursing Research within the National Institutes of Health that supported nursing outcomes research which has been published in leading interdisciplinary scientific journals and covered widely by the media.

The rise in nurses’ prospects in the USA was helped by the impact of the economic downturn on job loss in other sectors of the economy and the steady rise in nurses’ incomes to levels comparable to or exceeding comparable occupations like teaching. However, nursing’s popularity will not likely decline as the economy improves since the public is now better informed about the need for sufficient numbers of nurses, as evident by more than half of the 50 states passing some form of legislation or regulation to ensure safe hospital nurse staffing. Also worth mentioning in the context of Israel’s low nurse to doctor ratio, USA national workforce policy has constrained the growth of doctors because of concern about rapidly escalating healthcare expenditures. As a result, opportunities were created for nurses to successfully take on wider scope of practice and leadership in healthcare thus contributing to attractive career pathways for nurses and improved working conditions for all nurses.

Nursing is a profession that has traditionally offered substantial opportunities for social mobility, important for building a strong middle class. Investments in nursing also yield good value in terms of accessible, safe, effective, patient-centered health care, important to citizens in every country. Underinvestment in nursing is not in the public’s interest.

## Competing interests

The authors declare that they have no competing interests.

## Authors’ information

Linda H Aiken is the Claire M Fagin Leadership Professor of Nursing, Professor of Sociology, and Director of the Center for Health Outcomes and Policy Research, University of Pennsylvania School of Nursing in Philadelphia, Pennsylvania, USA. Matthew D McHugh is the Rosemarie Greco Term Endowed Associate Professor in Advocacy and Associate Director of the Center for Health Outcomes and Policy Research, School of Nursing, University of Pennsylvania.

## Commentary on

DeKeyser-Ganz F and Torren O: Israeli nurse practice environment characteristics, retention, and job satisfaction. Isr J of Health Policy Res 2013,3:5.
